# Extraction optimization and reactivity of 7α-acetoxy-6β-hydroxyroyleanone and ability of its derivatives to modulate PKC isoforms

**DOI:** 10.1038/s41598-024-67384-0

**Published:** 2024-07-23

**Authors:** Vera M. S. Isca, Gabrielle Bangay, Salvatore Princiotto, Lucília Saraíva, Daniel J. V. A. dos Santos, Alfonso T. García-Sosa, Patrícia Rijo

**Affiliations:** 1 CBIOS - Center for Research in Biosciences and Health Technologies, Universidade Lusófona, 1749-024 Lisboa, Portugal; 2 Research Institute for Medicines (iMed.ULisboa), Faculty of Farmacy, Universidade de Lisboa, 1649-003 Lisboa, Portugal; 3 Departamento de Ciencias Biomédicas (Área de Farmacología), Nuevos agentes antitumorales, Acción tóxica sobre células leucémicas, Facultad de Farmacia, Universidad de Alcalá de Henares, Ctra. Madrid-Barcelona Km. 33, 600 28805 Alcalá de Henares, Madrid, Spain; 4 LAQV/REQUIMTE, Laboratório de Microbiologia, Departamento de Ciências Biológicas, Faculdade de Farmácia, Universidade do Porto, 4050-313 Porto, Portugal; 5 Institute of Chemistry, University of Tartu, 54011 Tartu, Estonia; 6 Department of Food, Environmental and Nutritional Sciences (DeFENS), University of Milan, via Celoria 2, 20133 Milan, Italy

**Keywords:** Cancer, Computational biology and bioinformatics, Drug discovery, Molecular medicine, Oncology

## Abstract

Protein kinase C is a family of kinases that play important roles in carcinogenesis. Medicinal plants from *Plectranthus* spp. (Lamiaceae) are a well-known source of interesting abietanes, such as 7α-acetoxy-6β-hydroxyroyleanone (**Roy**). This study aimed to extract and isolate **Roy** from *P. grandidentatus* Gürke, comparing two extraction methods (CO_2_ supercritical and ultrasound-assisted acetonic extraction), and design new royleanone derivatives for PKC modulation focusing on breast cancer therapy. The concentration of **Roy** in the extracts was determined by HPLC–DAD. The supercritical extraction method yielded 3.6% w/w, with the presence of 42.7 μg mg^−1^ of **Roy** (yield of 0.13%), while ultrasound-assisted acetonic extraction yielded 2.3% w/w, with the presence of 55.2 μg mg^−1^ of **Roy** (yield of 0.15%). The reactivity of **Roy** was investigated aiming at synthetizing new ester derivatives through standard benzoylation and esterification reactions. The benzoylated (**Roy-12-Bz**) and acetylated (**Roy-12-Ac**) derivatives in the C12 position were consistently prepared with overall good yields (33–86%). These results indicate the 12-OH position as the most reactive for esterification, affording derivatives under mild conditions. The reported di-benzoylated (**RoyBz**) and di-acetylated (**RoyAc**) derivatives were also synthesized after increasing the temperature (50 °C), reaction time, and using an excess of reagents. The cytotoxic potential of **Roy** and its derivatives was assessed against breast cancer cell lines, with **RoyBz** emerging as the most promising compound. Derivatization at position C-12 did not offer advantages over di-esterification at positions C-12 and C-6 or over the parent compound **Roy** and the presence of aromatic groups favored cytotoxicity. Evaluation of royleanones as PKC-α, βI, δ, ε, and ζ activators revealed **DeRoy**’s efficacy across all isoforms, while **RoyPr** showed promising activation of PKC-δ but not PKC-ζ, highlighting the influence of slight structural changes on isoform selectivity. Molecular docking analysis emphasized the importance of microenvironmental factors in isoform specificity, underscoring the complexity of PKC modulation and the need for further exploration.

## Introduction

Cancer remains a leading cause of death worldwide, despite progress in developing new therapeutic approaches. The inherent toxic effects of anticancer chemotherapeutic drugs on the host demand that cancer therapy be based on more selective molecules, to avoid toxicity on non-tumoral cells^1^. Protein kinase C (PKC) is an attractive target for cancer therapy^2^ and comprises a family of serine-threonine kinases that control various cellular processes, including apoptosis, survival, differentiation, proliferation, and migration^3^. PKC family includes 10 isoforms, encoded by nine functionally and structurally related genes, that are classified as classical (α, βI, βII and γ), novel (δ, ε, η, φ), and atypical (ζ and λ\ι) PKCs^3,4^. PKC isoforms are composed of a conserved *N*-terminal regulatory region (C1 and C2 domain), and a *C*-terminal catalytic region (ATP binding and phosphotransferase activity)^5^. PKCs are activated by endogenous calcium, diacylglycerol (DAG), phospholipids and/or phorbol esters (PS)^6^. PS bind to the C1 domain and represent a competitive ligand for the physiological substrate DAG^7^. Conversely, atypical PKCs are structurally and functionally distinct from other PKCs, with a single C1 domain unresponsive to DAG/phorbol and lacking a C2 domain^8^. Nonetheless, PKC-ζ can be activated by lipid components such as arachidonic acid and ceramide^9^. It is generally accepted that most PKC activators bind to the regulatory domain^3^. Thus, the C1 domain has become the most attractive target for the design of selective PKC activators^6,7,10^. On the other hand, PKC inhibitors may interact with PKC at different sites of the protein^11–15^.

Members of the PKC family were originally categorized as oncogenes; however, individual isoforms exhibit context-dependent activities and may have distinct or even opposite roles in the regulation of cellular processes involved in cancer development. This complexity poses challenges in designing selective PKC modulators^8,16,17^. Moreover, PKCs share high sequence homology and structural similarity among themselves and with other kinases^2,8,10^, leading to limited availability of selective modulators^5,6^. In this context, PKC-α has deserved increased attention in breast cancer research. Its role is intricate, as it can function as both an anti-proliferative agent and a growth stimulant depending on the context^18^. While PKC-α generally protects cancer cells^2^, studies have shown that its activation with phorbol 12-myristate 13-acetate (PMA) inhibits epidermal growth factor-induced cell spreading and chemotaxis in MDA-MB-231 cells^19^. Additionally, overexpression of PCK-α in MCF-7 breast cancer cells alters the expression of other PKC isoforms and decreases estrogen receptor (ER) levels^8,20^, potentially contributing to the switch from ER-positive to ER-negative state^20^. On the other hand, downregulation of PKC-α is associated with invasive ductal carcinoma^21^ and is observed in epithelial cells of advanced breast tumors^22^.

**Figure 1:**
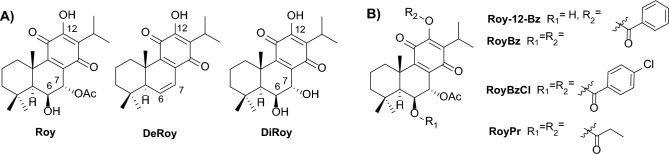
**A)** Natural royleanones from *Plectranthus* spp.: 7α-acetoxy-6β-hydroxyroyleanone (**Roy**), 6,7-dehydroroyleanone (**DeRoy**) and 6β,7α-dihydroxyroyleanone (**DiRoy**). **B)** Semi-synthetic derivatives previously prepared from **Roy**: 7α-acetoxy-6β-hydroxy-12-*O*-benzoylroyleanone (**Roy-12-Bz**), 6β-benzoyloxy-12-*O*-benzoylroyleanone (**RoyBz**), 7α-acetoxy-6β-(4-chloro)benzoyloxy-12-*O*-(4-chloro)benzoylroyleanone (**RoyBzCl**) and α-acetoxy-6β-propanoyloxy-12-O-propanoylroyleanone (**RoyPr**).

The isolation of new natural products provides novel scaffolds with potential biological activities for cancer therapy. Abietane diterpenoids, widely distributed in the Lamiaceae family, offer a broad spectrum of biological activities, including anti-inflammatory, antimicrobial, and antitumor activities^23^. *Plectranthus* spp. (Lamiaceae) are recognized sources of such compounds, with royleanone, coleon, and parvifloron-type diterpenoids showing notable medicinal properties, particularly antiproliferative effects.^23,25,26^ Among these, compound 7α-acetoxy-6β-hydroxyroyleanone (**Roy**, Fig. 1A) can be isolated in high yields from *Plectranthus grandidentatus* Gürke^24^ and has shown cytotoxicity against several types of human cancer cell lines (MCF-7, NCI-H460, SF-268, TK-10, UACC-62)^25^, alongside significant antimicrobial^26,27^ and immunomodulatory effects^28^. Similarly, the diterpene abietane 7-dehydroroyleanone (**DeRoy**, Fig. 1A), the major compound of the essential oil of *Plectranthus madagascariensis* Pers. Benth^29^, exhibits promising antioxidant, antimicrobial, and cytotoxic activities^29,30^. Another interesting example is the abietane diterpenoid 6β,7α-dihydroxyroyleanone (**DiRoy**, Fig. 1A), that can be isolated from *P. grandidentatus*, and *P. madagascariensis*, and which showed promising cytotoxic^25^ and antimycobacterial activities^31^.

Previous data from our research group^5,32^ have highlighted **Roy** and **DeRoy** as promising leads for future drug development with the ultimate purpose of enhancing their cytotoxic properties by exploiting the hydroxyl residues suitable for derivatizations (Fig. 1A). To date, several derivatives of **Roy** and **DeRoy** have been described^27,33–37^, although stability problems have been encountered in certain synthetic pathways, particularly in the synthesis of isocyanates, carbamates, and other products following the Mitsunobu reaction protocol. Overall, ester derivatives displayed promising stability and isolated yields^33^. Among these, examples include the patented derivative 7α-acetoxy-6β-benzoyloxy-12-*O*-benzoylroyleanone (**RoyBz**, Fig. 1B)^36^, the 7α-acetoxy-6β-hydroxy-12-*O*-benzoylroyleanone (**Roy-12-Bz**, Fig. 1B)^33^, the 7α-acetoxy-6β-(4-chloro)benzoyloxy-12-*O*-(4-chloro)benzoylroyleanone (**RoyBzCl**, Fig. 1B)^27,37^ and the 7α-acetoxy-6β-propanoyloxy-12-*O*-propanoylroyleanone (**RoyPr**, Fig. 1B)^27,37^. The antitumoral activity of the derivative **RoyBz** was extensively evaluated in colon cancer cell lines. It was the first PKC-δ-selective activator reported with strong antitumor effects demonstrated through pro-apoptotic and anti-migratory effects in colon cancer cells and xenograft mouse models, without any observed side toxicity. Additionally, preliminary molecular docking studies provide deeper insights into the interaction between **RoyBz** and the C1 domain of the PKC-δ isoenzyme’s *N*-terminal regulatory domain. These results suggested that abietanes could represent a novel class of PKC-δ selective modulators with potential therapeutic applications in colon cancer^5^. In the same way, the cytotoxic potential of the derivatives **RoyBzCl** and **Roy-12-Bz** was evaluated, revealing promising P-gp inhibition activity with IC_50_ values in the µM range^38^. However, to the best of our knowledge, these derivatives were not evaluated as PKC modulators for breast cancer therapy. Thus, this study aims to evaluate the effectiveness of royleanones as PKC activators within the context of breast cancer therapy.

## Results and discussion

### Roy extraction and isolation

The biological significance of **Roy** has already been demonstrated^25–28^, and its isolation from *P. grandidentatus* has been optimized^24^. Nevertheless, our goal was to conduct a comprehensive analysis of the advantages and disadvantages of CO_2_ supercritical extraction and ultrasound-assisted methods. For this phytochemical study the aerial parts of *P. grandidentatus* were used. The supercritical extraction method allowed the obtainment of one extract with 3.6% w/w of yield, while the acetonic extraction afforded an extraction yield of 2.3% w/w. Both extracts were very similar when compared by thin layer chromatography (TLC) and high-performance liquid chromatography (HPLC). The HPLC chromatograms provided a detailed and precise assessment, leading to the decision to combine the extracts. The crude extract was further fractionated by flash liquid chromatography using CH_2_Cl_2_ as eluent, resulting in three crude fractions that were compared with one sample of **Roy** by TLC (eluent Hex: AcOEt 90:10, 80:20, or 70:30). The fraction containing **Roy** (A1) was subsequently purified by dry flash chromatographic column using CH_2_Cl_2_ as eluent, resulting in five new fractions. Four of these fractions contained **Roy** and were subjected to sequential dry flash chromatographic columns using mixtures of Hex: AcOEt and AcOEt: MeOH as eluents. Some of the final fractions yielded **Roy**, obtained as pure yellow crystal plates by recrystallization from *n*-Hexane (Hex). The molecular structure was confirmed by NMR spectroscopy and compared with data from the literature^24^.

### Roy quantification by HPLC–DAD

The amount of **Roy** in acetonic and CO_2_ supercritical extracts was quantified by HPLC–DAD. The HLPC method used had been previously optimized by Bernardes et al.^24^ Extracts were analyzed at a concentration of 5 mg mL^−1^. One aliquot of 20 µL of each extract was injected in triplicate and the amount of **Roy** was calculated with resource to one linear calibration curve. In order to detect **Roy** with good sensitivity, 270 nm was selected as the detecting wavelength for the analysis, according to the λ max of **Roy** UV spectra. Temperature was not controlled. After settling the analytical conditions, the HPLC method was evaluated for several validation characteristics such as specificity, linearity, limits of detection (LOD), and quantification (LOQ).

Specificity was demonstrated by the peak purity of **Roy**. No interferences were observed at **Roy**’s retention time, indicating that the peak corresponded to pure **Roy**. Linearity was determined through the analyses of four concentrations of the analyte, and the correlation between observed peak areas and respective concentration afforded the linear calibration graphic. Regression analyses showed good linearity, with R^2^ of 0.99. The retention time (Rt), regression equation, correlation coefficient (R^2^), and LOD and LOQ values, obtained for **Roy** are presented in Table 1.

**Table 1:** Parameters used for the calibration curve.

Rt/min	Regression equation	Correlation coefficient (R^2^)	LOD/ng mL^−1^	LOQ/ng mL^−1^
11.9	y = 4,769,127 x + 527,332	0.9981	0.20	0.60

The identification of **Roy**´s peaks in each chromatogram of the extract was achieved through the retention time and the UV spectra analysis. The values obtained were expressed as means ± SD (at least n = 3). In this work, a total of 3.1 ± 0.05 g of **Roy** was calculated in both extracts. The CO_2_ supercritical extract revealed the presence of 42.7 ± 0.7 μg mg^−1^ (yield of 0.13%), while the ultrasound-assisted acetonic extract showed 55.2 ± 1.0 μg mg^−1^ of **Roy** (yield of 0.15%). These results indicated that acetonic extraction was slightly more effective than the CO_2_ supercritical extraction. Further, it has been taken under consideration that CO_2_ supercritical extraction requires higher costs due to the need for CO_2_, dedicated equipment and training, making it more time-consuming and economically unfavorable. Conversely, acetonic extraction offers simplicity, speed, and higher yield, making it a more attractive option. Although acetonic extraction requires a significant amount of solvent, acetone can be recovered after evaporation and reused for other applications.

The amount of **Roy** present in the CO_2_ supercritical extract was slightly lower than that reported by Bernardes et al., (57 μg mg^−1^)^24^. However, our study showed a significantly higher yield of **Roy** in ultrasound acetonic extract (55.2 μg mg^−1^) compared to the previous report (8.04 μg mg^−1^)^24^. This significative difference can be attributed to variations in the ultrasound extraction parameters. While Bernardes et al. reported a single ultrasound-assisted acetonic extraction of 60 min, our study employed three cycles of 30 min each. Therefore, a higher amount of **Roy** was extracted from the plant when multiple cycles of limited duration were performed. This optimization of the acetonic extraction method resulted in a higher extraction efficiency, demonstrating the importance of process optimization for maximizing yield.

### Roy reactivity

**Figure 2:**
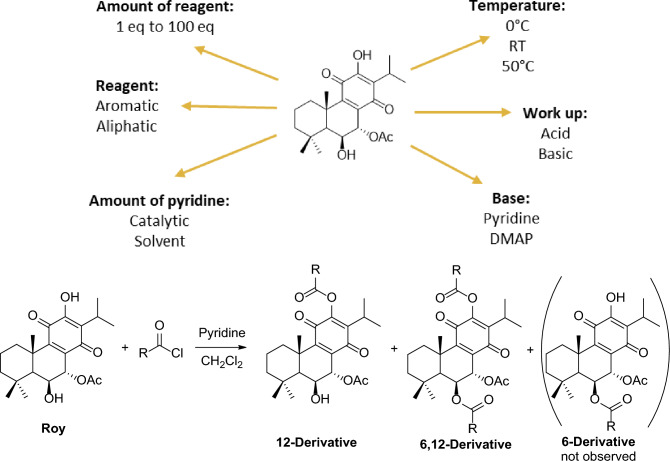
Reaction scheme and conditions tested for esterification reactions of **Roy**.

The preparation of derivatives from royleanones has been well described, with ester derivatives proving to be stable^27,33–37^. Hence, we investigated the esterification of **Roy** using benzoyl chloride (Table 2) and acetic anhydride (Table 3) as reagents. To determine the optimal reaction conditions, we explore several parameters, including the quantities of reagents, base, catalyst, temperature, and work up conditions (Fig. 2).

**Table 2:** Reactional conditions tested for esterification with benzoyl chloride^a^.


Entry	Benzoyl Chloride (eq)	Solvent		Catalyst		Temp. (°C)	Time (min)	Product/yield^b^ (%)
		CH_2_Cl_2_ (mL)	Pyridine (mL)	Pyridine (eq)	DMAP (eq)			
1	15	2	–	18	–	rt	60	**Roy-12-Bz** 69%
2	4	2	–	6.5	–	0	60	**Roy-12-Bz** 58%
3	4	2	–	2.5	–	25	5	**Roy-12-Bz** 36%
4	100	–	1	–	1	25	Overnight	**Roy-12-Bz** n.i
5	100	–	1	–	1	50	120	**RoyBz** 28%
6	100	–	0.5	–	–	50	Overnight	**RoyBz** 79%
7	8	–	0.5	–	–	25	5	**Roy-12-Bz** n.i
8	8	–	0.5	–	–	50	30	**Roy-12-Bz** 42%
9	17	–	0.5	–	–	50	12 h	**RoyBz** 50%

^a^The reactions were carried out with 10 mg of **Roy**. ^b^Isolated Yields. n.i., not isolated.

**Table 3:** Reactional conditions tested for esterification with acetic anhydride^a^.


Entry	Acetic anhydride (eq)	Pyridine (mL)	Temp. (°C)	Time (min)	Work-up	Product/yield^b^ (%)
1	1	0.5	rt	15	No	**Roy-12-Ac** 77%
2	11	0.5	rt	Overnight	No	**Roy-12-Ac** 65%
3	100	1	50	Overnight	No	**Roy-12-Ac** 50% + **RoyAc** 48%
4	8	0.5	0	15	No	**Roy-12-Ac** 86%
5	1	0.5	0	15	No	**Roy-12-Ac** 57% + **Roy** (decomposition)
6	1	0.5	0	15	Acidic (5% HCl)	**Roy-12-Ac** 33% + **Roy** (decomposition)
7	1	0.5	0	15	Basic (5% NaOH)	**DiRoy** 91%

^a^The reactions were carried out with 3 to 5 mg of **Roy**. ^b^Isolated Yields.

Under the reaction conditions reported in Table 2, benzoylation of **Roy** always afforded two different products, namely, **Roy-12-Bz** and **RoyBz**. Both compounds were obtained as yellow powder, which tends to crystallize in *n*-hexane and whose structures were confirmed by NMR spectroscopy and compared with the literature^34,38,39^. Curiously, it was not possible to obtain the mono-benzoylated product in position C-6 (Fig. 2) following this protocol, and to our knowledge, the preparation of benzoylated derivative at C-6 has not been achieved.

The number of equivalents needed, and the addition rate of the reagent were evaluated. The reagent was portion wise (or dropwise) added to the reaction. Dropwise addition of 15 eq of benzoyl chloride led to the formation of **Roy-12-Bz** in 1 h, with 69% of isolated yield. The same conditions were tested at 0 °C, but no significant changes were observed. Conversely, when the excess of benzoyl chloride was added at once, the reaction was very fast, and all the starting material **Roy** was consumed in 5 min, affording the same derivative in 36% isolated yield. Heating up to 50 °C and using pyridine as the solvent facilitated the formation of **Roy-12-Bz** in 30 min, further confirming the high reactivity of C-12-OH towards the benzoylation.

For the successful preparation of **RoyBz** in good yields (50–79%), the reaction mixture was left to react overnight with an excess of all reactants at 50 °C. Lower yields (28%) were obtained when DMAP was added in presence of an excess of the reactants and heated up to 50 °C for 2 h.

Similar reactivity was observed for the reaction with acetic anhydride. Both 7α-acetoxy-6β-hydroxy-12-*O*-acetylroyleanone (**Roy-12-Ac**) and 7α,6β-diacetoxy-12-*O*-acetylroyleanone (**RoyAc**) were successfully prepared, but the synthesis of the 6-OH derivative was not achieved (Fig. 2). Both compounds were isolated as yellow powder, with a tendency to crystallize in Hex. The structure of **RoyAc** was confirmed by NMR analysis and compared with literature data^35^. **Roy-12-Ac** has been synthesized for the first time in this study, and full structural characterization has been reported herein.

At room temperature, the derivative **Roy-12-Ac** was obtained in 15 min using one equivalent or small excess of acetic anhydride with overall good yields (65–77%). Adding one equivalent of acetic anhydride at 0 °C resulted in the total consumption of **Roy** within 15 min (as observed by TLC), but subsequent degradation occurred during the isolation process, yielding a small amount of starting material alongside a lower yield of **Roy-12-Ac** (57%). However, the use of a small excess of acetic anhydride at 0 °C led to the highest isolation yield for **Roy-12-Ac** (87%). On the other hand, to obtain **RoyAc** the reaction mixture was allowed to react overnight with an excess of all reactants at 50 °C. Further purification of the resulting crude allowed the isolation of two products, namely, monofunctionalized **Roy-12-Ac** (50% yield) and desired **RoyAc** (48% yield).

Optimization of the work-up conditions was attempted based on previous hemi-synthetic studies^35^. The treatment of the reaction mixture with a 5% solution of HCl, intended to neutralize the pyridine in the reaction environment, led to increased decomposition and consequently worse **Roy-12-Ac** isolation yield (33%). Stopping the reaction with a 5% solution of NaOH to quench residual acetic anhydride caused full decomposition of the product and cleavage of the acetoxy group in position C-7, resulting in the tri-hydroxylated product 6β,7α-dihydroxyroyleanone (**DiRoy**), isolated in 91% yield as yellow powder, which tended to crystallize in Hex. The structure of **DiRoy** was confirmed by NMR spectroscopy and compared with the literature^35,40^. Therefore, treatment of the crude mixture with even weak acidic or alkaline solutions led to the degradation of the desired product through ester hydrolysis.

### Effect of the derivatives in human breast adenocarcinoma cell lines

The cytotoxic activity of natural royleanones **Roy** and **DeRoy**, and synthetized derivatives **DiRoy**, **RoyBz**, **Roy-12-Bz**, **RoyAc**, **Roy-12-Ac**, and **RoyPr**, was thoroughly investigated in breast cancer cell lines (MCF-7, MDA-MB-231, MDA-MB-468) and against non-tumorigenic fibroblasts HFF-1 cells, through the sulforhodamine B (SRB) assay^5^. The cytotoxic effect (IC_50_ values, Table 4) was determined from dose–response curves.

**Table 4:** Cytotoxicity of the derivatives against human breast adenocarcinoma cell lines. Inhibitory concentration (IC_50_), reported in µM, was based on dose–response curves.

Compound	MCF-7	MDA-MB-231	MDA-MB-468	HFF-1
Roy	2.09 ± 0.42	0.35 ± 0.12	0.30 ± 0.16	1.02 ± 0.28
DeRoy	60.44 ± 3.69^41^	nt	nt	nt
DiRoy	> 20	> 20	> 20	nt
RoyBz	1.66 ± 0.10	1.50 ± 0.85	1.40 ± 0.08	3.58 ± 0.27
Roy-12-Bz	4.32 ± 0.24	1.65 ± 0.15	1.28 ± 0.00	1.16 ± 0.22
RoyAc	4.25 ± 0.57	1.86 ± 0.09	3.25 ± 0.20	4.97 ± 0.34
Roy-12-Ac	5.67 ± 0.24	2.43 ± 0.15	1.60 ± 0.16	0.60 ± 0.04
RoyPr	28.3 ± 2.4^42^	nt	nt	nt
Doxo	0.60 ± 0.01	1.2 ± 0.05	0.30 ± 0.04	0.10 ± 0.12

nt, not tested; Doxo, Doxorubicin.

To explore breast cancer biology and screening for new anti-tumoral agents, widely used cell lines MCF-7 and MDA-MB-231/MDA-MB-468 offer distinct characteristics. MCF-7 cells, being ER-positive, PR-positive, and HER2-negative, exhibit slower growth and less aggressiveness. In contrast, triple-negative MDA-MB-231/MDA-MB-468 cells are more aggressive, invasive, and possess higher metastatic potential^43^. The cytotoxic effects of **Roy**^25^, **DiRoy**^25^, **RoyBz**^42^, and **RoyAc**^42^ against MCF-7 cell line were previous evaluated using the MTT colorimetric method. Overall, the results obtained in this study through the SRB assay remain previous. **Roy** displayed significant anti-proliferative activity against all cell lines tested (IC_50_ of 2.09 µM, 0.30 µM, 0.35 µM and 1.02 µM on MCF-7, MDA-MB-231, MDA-MB-468, and HFF-1, respectively). A compound with cytotoxic effects on normal cells can have significant side effects and safety concerns^44^, thus chemical modifications can be crucial role to improve selectivity towards cancer cells. All derivates were active against the tested cancer cell lines with the analogue **RoyBz** emerging as the most promising derivative with lower IC_50_ values (IC_50_ of 1.66 µM, 1.50 µM and 1.40 µM on MCF-7, MDA-MB-231 and MDA-MB-468, respectively) and selectivity against cancer cell lines (IC_50_ of 3.58 µM on HFF-1 cell line). Additionally, **RoyBz** was the only derivative to surpass the cytotoxic activity of the parent compound **Roy** in the cell line MCF-7. These results suggest that derivatization on position C-12 does not confer any advantage compared to derivatization at both positions or when compared to the parent compound **Roy**. Additionally, it appears that aromatic groups favor the cytotoxicity against the MCF-7 cells (IC_50_ of 1.66 µM for **RoyBz**), compared to aliphatic chains (IC_50_ values of 4.45 µM for **RoyAc**, and 28.3 µM for **RoyPr**^42^). On the other hand, **DiRoy**, a royleanone structure with three hydroxyl groups, and **DeRoy**, the structure with a double bound in the positions C-6 and C-7, exhibit a significant decrease in the cytotoxic effects, as IC_50_ values above 20 μM suggest a weak cytotoxicity^45^, supporting the hypothesis that the acetoxy group at position C-7 may play a crucial role in cytotoxic activity against breast cancer cell lines.

### Effect of compounds on individual PKC isoforms

PKC isoforms are key targets for cancer therapy due to their significant roles in cancer development and progression. The selective-PKC-δ activation observed for **RoyBz** is well established^5^. Nonetheless, it is important to understand the effect of **Roy** on PKC isoforms. Due to the specificity of the assay, it was decided to evaluate only few compounds: the natural ones **Roy** and **DeRoy** and two derivatives, one aromatic ester **RoyBz** and one aliphatic ester **RoyPr**^27^ as PKC-α, -βI, -δ, -ε, and -ζ activators using the yeast PKC assay. The results can be consulted in Table 5.

**Table 5:** EC_50_ values of compounds tested on individual PKC isoform, using the yeast PKC assay.

Compounds	EC_50_ (nM)				
	PKC-α	PKC-βI	PKC-δ	PKC-ε	PKC-ζ
**PMA**	111.6 ± 18.4	243.2 ± 69.1	573.8 ± 36.7	1678 ± 46.48	nt
**ARA**	nt	nt	nt	nt	205.4 ± 32.6
**DeRoy**	15 ± 1.9	0.97 ± 4.34	3.1 ± 0.60	5.8 ± 0.70	43.8 ± 2.32
**Roy**	350 ± 42	423 ± 67	*ND*	994 ± 63	4113 ± 159
**RoyBz**	*ND*	*ND*	107.53^5^	*ND*	*ND*
**RoyPr**	195 ± 16	229 ± 21	325 ± 49	770 ± 46	*ND*

For the determination of EC_50_ values were considered the concentration of compound that caused 50% of the maximal growth inhibition caused by the positive controls (phorbol 12-myristate 13-acetate, PMA, for cPKCs and nPKCs; arachidonic acid, ARA, for PKCζ), which was set as 100%. Data are mean ± SEM of four independent experiments. nt, not tested; ND, non-determinable (when the maximal response achieved was lower than 50% growth inhibition).

When analyzing the results (Table 5), **RoyBz** recorded a notable selective activity towards the PKC-δ isoform^5^. On the other hand, **Roy** activated all PKC isoforms except for PKC-δ. However, the obtained EC_50_ (half maximal effective concentration) values for this compound were not significant when compared to the controls used (PMA and ARA). Curiously, **DeRoy** displayed promising EC_50_ values on all the isoforms with increased potential comparing to PMA and ARA. Although not selective for a specific isoform, its efficacy to activate isoforms from the three PKC sub-families makes it a valuable positive control for PKC evaluation assays. The introduction of propanoyl groups (**RoyPr**) in **Roy**, led to an overall decrease of the EC_50_ values. Interestingly, **RoyPr** seems to be also an activator of PKC-δ, but not of PKC-ζ. In a similar manner to PMA, **RoyPr** only activates the classical and novel PKC isoforms. These results suggest that slight changes in the structure affect the selectivity to each isoform. These preliminary results promote further studies, with new esters, to better understand the selectivity to PKC isoforms.

To get more insights on the origins of selectivity and activity, a molecular docking strategy was used based on our previous results using AutoDock^5,46^ that provided an explanation for the activity of **RoyBz** in PKC-δ, by showing similar interaction patterns between this molecule and 13-acetylphorbol (PRB; positive control; c.f. Figure 1 of Bessa et al.^5^). To focus on breast cancer, the research was centered on PKC-α. Taking as example the PKC-δ and PKC-α isoforms, the difference in the PRB binding region is due to M239G, W252Y, V255I, and K256H. Although only the first two are immediately next to PRB, the sidechains face away. However, these mutations can probably change the lipid/water microenvironment around this binding site. Therefore, for a closer comparison, instead of using the 2ELI NMR structure (available in Protein Data Bank^47^), the PKC-δ 1PTR X-ray crystal structure was mutated and reverted to the PKC-α isoform.

**Figure 3:**
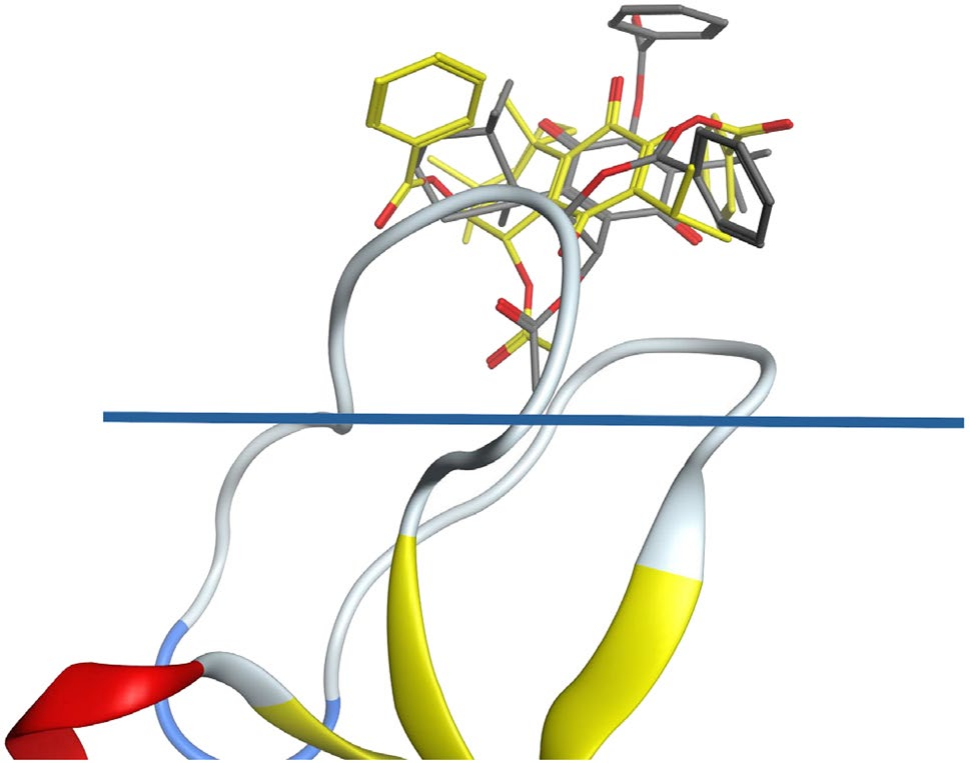
Top ranked docking poses for **RoyBz** in the α and δ isoforms (gray and yellow carbons, respectively). The blue line marks the position where the phospholipid carbon tails begin insertion in the membrane, according to OPM.

The top-ranking poses of **RoyBz** in both α and δ isoforms are depicted in Fig. 3. As observed, although both poses have the same OAc group inserted in the groove, the **RoyBz** pose in the variant has a benzoyl group protruding with the carbonyl pointing to the middle of the membrane. Although the free energy of binding for **RoyBz** in PKC-α is reduced from − 7.87 to − 6.38 kcal mol^−1^, it is not enough to explain the inactivity with the α isoform and selectivity towards δ (PRB has an interaction of − 7.54 kcal mol^−1^ with δ). However, according to the Orientations of Proteins in Membranes (OPM) database information^48^, the PRB molecule sits inside the molecular membrane on the cytoplasmic inner leaflet. Taking this into consideration and the fact that **RoyBz** has the benzoyl group located in a region inappropriate for favorable interactions with the lipid tails, and considering that these facts are not accounted in the docking evaluation, this could explain the differences found between variants. This means that not only the predicted docking score needs to be considered, but also the capacity of the molecules to partition in the bilayer and the possible changes in the nature of the amino acids producing also changes in the microenvironment hydrophilic/lipophilic balance.

Overall, the antitumoral effect of **Roy**, its analogues, and **DeRoy** was investigated in breast cancer cells, with a focus on evaluating their impact on PKC isoforms, which are pivotal targets in cancer therapy. The study of these royleanones revealed significant cytotoxic activity against breast cancer cell lines. **Roy** exhibited potent anti-proliferative effects across all tested cell lines. The importance of selective cytotoxicity against cancer cells to minimize off-target effects was underscored by the superior activity of **RoyBz**, which demonstrated lower IC_50_ values and greater selectivity towards cancer cells compared to normal fibroblasts. Furthermore, the activation profile of PKC isoforms by royleanone derivatives revealed distinct effects. **RoyBz** selectivity activated PKC-δ, a critical target in colon cancer therapy due to its role in cancer progression^5^. **Roy**, **DeRoy** and **RoyPr** exhibited varying activation profiles across multiple PKC isoforms. The introduction of propanoyl groups in **RoyPr** affect its activity in the cell MCF-7 line and altered its isoform selectivity, suggesting that structural modifications can influence both cytotoxicity and PKC activation profiles.

These findings underscore the complex interplay between chemical structure, cytotoxic efficacy, and isoform-specific PKC activation, guiding future research towards optimizing royleanone derivatives for targeted cancers that are positively influenced by the modulation of PKC isoforms, particularly in breast cancer treatment. Further exploration of new derivatives and their effects on PKC isoforms is warranted to refine their therapeutic potential and elucidate their specific mechanisms of action in cancer cells. This exploration may lead to the discovery of promising PKC modulators with high potency and isoform-selectivity within this compound family.

## Methods

### Plant material

The plant material, *P. grandidentatus* Gürke was cultivated in Parque Botânico da Tapada da Ajuda (Instituto Superior Agrário, Lisbon, Portugal) from cuttings obtained from the Kirstenbosch National Botanical Garden (Cape Town, South Africa). Voucher specimens (572/2008) were deposited in Herbarium João de Carvalho e Vasconcellos (ISA). The plant name has been checked with http://www.theplantlist.org^49^.

### Extraction and isolation

Acetone ultrasonic-assisted extraction was adapted from Bernardes C.E.S. et al.^24^ The leaves and steams of *P. grandidentatus* were air dried. 2.334 kg of the dried plant was grinded to powder and with acetone (15 × 3.4 L), in an ultrasound equipment. The ultrasonic bath (Sonorex Super RK 510 H; Bandelin, Berlin, Germany) operated at room temperature, for 30 min, for 3 times, at 35 Hz with maximum input power of 320 W. Filtration and evaporation of the solvent (under vacuum, 40 °C) yielded a residue of 52.74 g (2.3% w/w).

Supercritical fluid extraction was carried out in an apparatus, equipped with a 500 cm^3^ sample cell, which had been previously described by Pereira et al.^50^ a sample of 130.11 g of powder plant was extracted with supercritical CO_2_ for 4 h at 40 °C and 230 bar, using a fixed CO_2_ flow rate of 0.3 kg h^−1^. The supercritical fluid extract was recovered by washing the collection vessel and tubing the expansion line with acetone. The solvent was subsequently removed in a rotary evaporator and yielded a residue of 4.67 g (3.6% w/w).

The isolation process was adapted from Bernardes et al.^24^ The crude extract was subjected to sequential liquid and dry flash chromatographic separations. The liquid flash chromatographic column used silica gel (Merck 9385) as stationary phase and CH_2_Cl_2_ as eluent. The dry flash chromatography used silica gel (Merck 9385) as stationary phase and mixtures of Hex: AcOEt and AcOEt: MeOH as eluents. The fractions obtained were compared with one sample of **Roy** by TLC (Eluent Hex: AcOEt 90:10, 80:20, or 70:30). **Roy** was obtained from recrystallization from Hex.

### Quantification of Roy by HPLC–DAD

The quantification of **Roy** in *P. grandidentatus* extracts was adapted from Matias et *al*. 2019^32^. The quantification was performed using HPLC–DAD and complementary spectroscopic methodologies. The quantification of the identified compound was carried out in a Dionex Ultimate 3000 UHPLC system with diode array detector (DAD; Thermo Fisher Scientific Inc. MA, USA), equipped with a Nucleodur 100–5 C18ec, (250 × 4.0 mm i.d., 5 µm) column, from Macherey–Nagel and Thermo Scientific™ Chromeleon™ 7.3 Chromatography Data System software (Thermo Fisher Scientific Inc. MA, USA). Each sample was analysed (after 20 µL injection) and a gradient elution mixture composed of solution A (methanol), solution B (acetonitrile), and solution C (0.3% trifluoroacetic acid in water) was used as follows: 0 min, 15% A, 5% B, and 80% C; 10 min, 70% A, 30% B, and 0% C; 25 min, 70% A, 30% B, and 0% C; 28 min, 15% A, 5% B, and 80% C; and 31 min, 15% A, 5% B, and 80% C. The flow rate was set at 1 mL min^−1^. Compound identification was based on retention time. The time of analysis was 31 min, including the stabilization of the RP-18 column. For quantification and identification purposes, **Roy** was detected using chromatograms corresponding to 270 nm, and its content in the plant extracts was estimated from the peak areas based on a calibration curve obtained with an authentic standard of **Roy**. All analyses were performed in triplicate. LOD and LOQ were assessed based on Signal-to-Noise approach^51^.

### Synthesis general procedure

For the general procedure, **Roy** (around 20 μmol) was dissolved in of dichloromethane (DCM, 2 mL) or pyridine (0.5–1 mL) with stirring (400 rpm) in a 5 mL round bottom flask, at room temperature, under heating or in an ice bath. When DCM is used as solvent, excess of pyridine was added (2.5–18 eq). Then, benzoyl chloride (or acetic anhydride) (1–100 eq) was added to the reaction flask. Reactions were followed by TLC (eluent DCM: Acetone, 98:2) until total consumption of **Roy**, then concentrated under reduced pressure. Products purification was performed by preparative chromatography, using as the eluent a mixture of CHCl_3_:Acetone (99:1) to purify **RoyBz** and CH_2_Cl_2_:Acetone (99:1) for **Roy-12-Bz**, **Roy-12-Ac** and, **RoyAc.** The purity of the compounds was assessed by HPLC, according to the methodology described above. All compounds exhibited a purity exceeding 95%.

### Compounds characterization

**Roy (7α-acetoxy-6β-hydroxyroyleanone)**: Yellow crystals. ^1^H-NMR (300 MHz, Chloroform-*d*, ppm): δ 7.22 (s, 1H, 12-OH), 5.66 (dd, J = 2.2, 0.7 Hz, 1H, H-7β), 4.31 (s, 1H, H-6α), 3.16 (sept, *J* = 7.1 Hz, 1H, H-15), 2.63 (d, *J* = 12.8 Hz, 1H, H-1β), 2.04 (s, 3H, Me-7α-OAc), 1.89–1.78 (m, 1H, H-2β), 1.61 (s, 3H, Me-20), 1.55–1.46* (m, 2H, H-2α and H-3β), 1.33 (s, 1H, H-5α), 1.23* (s, 3H, Me-19), 1.22* (s, 3H, Me-17), 1.21* (s, 1H, H-3α^+^), 1.20* (s, 3H Me-16), 1.18* (s, 1H H-1α^+^), 0.94 (s, 3H, Me-18) ppm. ^13^C-NMR (75 MHz, Chloroform-*d*, ppm): δ 185.91 (C11), 183.40 (C14), 169.83 (7α-COCH_3_), 151.04 (C12), 150.04 (C9), 137.19 (C8), 124.76 (C13), 68.86 (C7), 67.06 (C6), 49.86 (C5), 42.39 (C3), 38.75 (C10), 38.55 (C1), 33.80 (C18), 24.28 (C15), 23.94 (C19), 21.60 (C20), 21.08 (7α-COCH_3_), 19.97 (C16), 19.84 (C17), 19.10 (C2).

**RoyBz (7α-acetoxy-6β-benzoyloxy-12-*O*-benzoylroyleanone)**: Yellow crystals, 79%. ^1^H-NMR (300 MHz, Chloroform-*d*, ppm): δ 8.16 (d, *J* = 7.3 Hz, 2H, H-23), 8.00 (d, *J* = 7.4 Hz, 2H, H-28), 7.69 (t*, J* = 7.4 Hz, 1H, H-30), 7.61–7.57 (m, 1H, H-25), 7.54 (t, *J* = 7.4 Hz, 2H, H-29), 7.43 (t, *J* = 7.3 Hz, 2H, H-24), 5.90 (d, *J* = 1.9 Hz, 1H, H-7β), 6.37 (s,* J* = 1.9 Hz, 1H, H-6α), 3.19 (sept, *J* = 7.0 Hz, 1H, H-15), 2.67–2.53 (m, 1H, H-1β), 2.11 (s, 3H, Me-7α-OAc), 1.84–1.82 (m, 1H, H-2β), 1.78 (s, 3H, Me-20), 1.64–1.55* (m, 2H, H-2α and H-5α), 1.51–1.46 (m, 1H, H-3β), 1.42–1.36 (m, 1H, H-3α), 1.33–1.22* (m, 7H, H-1α, Me-16 and Me-17), 1.07 (s, 3H, Me-18), 1.00 (s, 3H, Me-19).

**Roy-12-Bz (7α-acetoxy-6β-hydroxy-12-*O*-benzoylroyleanone)**: Yellow amorphous solid, 69%. mp: 216–218 °C. $\left[ \upalpha \right]_{D}^{20} = \user2{ } + 33.1 ^\circ$ (*c* 0.151, CHCl_3_). IR ${\overline{\nu }}$_max_: 3476.8, 2971.5, 2935.7, 2873.7, 1745.8, 1667.6, 1605.7, 1540.5, 1455.7, 1371.0, 1224.3, 1142.8, 1113.4, 1061.3, 1015.6, 771.1, 751.6, 702.7 cm^−1^.

^1^H-NMR (300 MHz, Chloroform-*d*, ppm): δ 8.15 (d, *J* = 7.1 Hz, 2H, H-23), 7.72–7.61 (m, 1H, H-25), 7.52 (t, *J* = 7.6 Hz, 2H, H-24), 5.70 (d, *J* = 1.7 Hz, 1H, H-7β), 4.34 (s, 1H, H-6α), 3.19 (sept, *J* = 6.9 Hz, 1H, H-15), 2.52 (d, *J* = 9.4 Hz, 1H, H-1β), 2.07 (s, 3H, Me-7α-OAc), 1.88–1.71 (m, 1H, H-2β), 1.64 (s, 3H, Me-20), 1.60–1.52 (m, 1H, H-2α), 1.51–1.42 (m, 1H, H-3β), 1.38 (s, 1H, H-5α), 1.28–1.19* (m, 11H, Me-19, Me-17, H-3α, H-1α, Me-16), 0.95 (s, 3H, Me-18).

**RoyAc (7α,6β-diacetoxy-12-*O*-acetylroyleanone)**: Yellow amorphous solid, 48%. ^1^H NMR (300 MHz, Chloroform-*d*, ppm): δ 5.70 (d, J = 1.9 Hz, 1H, H-7β), 5.50 (s, 1H, H-6α), 3.19–3.02 (m, 1H, H-15), 2.55 (d, J = 13.0 Hz, 1H, H-1β), 2.35 (s, 3H, 12-OAc), 2.05 (s, 3H, 6β-OAc), 2.04 (s, 3H, 7α-OAc), 1.84–1.77 (m, 1H, H-2β), 1.61 (s, 3H, Me-20), 1.53–1.43* (m, 3H, H-2α, H-3β, H-5α), 1.28* (s, 2H, H-1α, H-3α), 1.19 (dd, *J* = 7.0 Hz, 6H, Me-16, Me-17), 1.00 (s, 3H, Me-19), 0.99 (s, 3H, Me-18).

**Roy-12-Ac (7α-acetoxy-6β-hydroxy-12-*O*-acetylroyleanone)**: Yellow oil, 87%. $\left[ \upalpha \right]_{D}^{20} = + 25.0 ^\circ$ (*c* 0.079, CHCl_3_). IR ${\overline{\nu }}$_max_: 2965.0, 2929.1, 2854.2, 1762.1, 1670.9, 1615.4, 1468.7, 1371.0, 1273.2, 1234.0, 761.4 cm^−1^. ^1^H NMR (300 MHz, Chloroform-*d*, ppm): δ 5.65 (d, *J* = 1.6 Hz, 1H, H-7β), 4.32 (s, 1H, H-6α), 3.11 (sept, *J* = 7.1 Hz, 1H, H-15), 2.51 (d, *J* = 13.3 Hz, 1H, H-1β), 2.34 (s, 3H, 12- OAc), 2.05 (s, 3H, 7α-OAc), 1.85–1.76 (m, 1H, H-2β), 1.63 (s, 3H, Me-20), 1.58—1.50* (m, 1H, H-2β), 1.46 (d,* J* = 14.2 Hz, 1H, H-3β), 1.34 (s, 1H, H-5α), 1.22* (s, 3H, Me-19), 1.21* (s, 3H, Me-17), 1.20* (s, 1H, H-3α^+^), 1.19* (s, 3H, Me-16), 1.18* (s, 1H, H-1α^+^), 0.94 (s, 3H, Me-18). ^13^C NMR (75 MHz, Chloroform-*d*, ppm): δ 185.94 (C-14), 179.90 (C-11), 169.83 (7αCOCH3), 168.44 (12-COCH3), 153.01 (C-9), 149.50 (C-12), 139.43 (C-13), 135.78 (C-8), 68.98 (C-7), 67.34 (C-6), 49.88 (C-5), 39.00 (C-10), 38.43 (C-1), 33.86 (C-4), 33.65 (C-18), 25.32 (C-15), 23.98 (12- COOCH_3_), 21.85 (C-19), 21.04 (C-20), 20.58 (7α-COCH3), 20.41 (C-16), 20.34 (C17), 19.03 (C2). HRMS (ESI–MS): m/z calculated for C_24_H_32_O_7_ [M + H]^+^ 433.2221, found 433.22179.

**DiRoy (7α,6β-dihydroxyroyleanone)**: Yellow needles, 92%. ^1^H NMR (300 MHz, Chloroform-*d*, ppm): δ 7.29 (s, 1H, 12-OH), 4.51 (s, 1H,H-7β), 4.46 (s, 1H, H-6α), 3.17 (hept, J = 7.1 Hz, 1H, H-15), 2.95 (s, 1H, H-1β), 2.60 (dt, J = 13.7, 3.3 Hz, 2H, H-1β), 1.92–1.76 (m, 1H, H-2β), 1.61 (s, 3H, Me-20), 1.58–1.31* (m, 3H, H-2α, H-3β, H-5α), 1.26 (s, 3H, Me-19), 1.24–1.20 (m, 8H, H-1α, H-3α, Me 16, Me-17), 1.05 (s, 3H, Me-18).

*overlapped signal, ^+^Interchangeable signals.

### Molecular docking

Molecular docking experiments were conducted in a similar way as our previous successful predictions for PKC-δ^5^, with AutoDock v4.2.6^46^. The 1PTR PKC-δ isoform structure was obtained from the PDB and the needed mutations (M239G, W252Y, V255I and K256H) performed in MOE^52^. The aminoacid protonation states were assigned using the Protonate 3D module within MOE and exported as PDB file. All tested molecules where built and energy minimized in MOE and their energy minimized using default parameters. The PBD files were converted to the respective PDBQT ligand or receptor files using python scripts available in MGLTools. Docking poses and interactions were visually inspected within MOE.

### Human cell lines and growth conditions

Royleanones and doxorubicin (Doxo) were tested on human breast adenocarcinoma lines. Human breast adenocarcinoma MCF‐7, MDA-MB-231 and MDA-MB-468, and non‐tumorigenic foreskin fibroblasts HFF‐1 cell lines were purchased from the ATCC (Rockville, MD, USA). Cancer cells were routinely cultured in RPMI‐1640 medium with UltraGlutamine from Biowest (VWR, Carnaxide, Portugal) supplemented with 10% FBS (fetal bovine serum) from Biowest (VWR). HFF‐1 cells were cultured in DMEM/F‐12 supplemented with 10% FBS. All cells were maintained at 37 °C in a humidified atmosphere of 5% CO_2_. Cells were routinely tested for mycoplasma infection using the MycoAlert™ PLUS mycoplasma detection kit (Lonza).

### Sulforhodamine B (SRB) assay

For the evaluation of the effect of the compounds on cell proliferation, the SRB assay was performed. Human cell lines were seeded in 96‐well plates at a density of 5.0 × 10^3^ (MCF-7, MDA-MB-231, and HFF-1) cells and 7.5 × 10^3^ (MDA-MB-468) cells per well and allowed to adhere for 24 h. Cells were treated for 48 h with serial dilutions of the compounds (ranging from 0.1 to 30.0 µM). The effects on cell proliferation were assessed by SRB assay, as described^5^, and IC_50_ values were determined for each cell line using the GraphPad Prism software version 7.0 (La Jolla, CA, USA).

### Yeast PKC screening assay

The royleanones **Roy**, **RoyBz** and **RoyPr** were tested on the yeast assay as reported in^3^. Briefly, *Saccharomyces cerevisiae* cells individually expressing mammalian PKC-α, -βI, -δ, -ε, or -ζ, and control yeast (transformed with empty vector) obtained in previous works were used. Cells were grown in galactose selective medium in the presence of 0.1—30 μM of compounds (or 0.1% DMSO only) for approximately 42 h. Yeast growth was analyzed by counting the number of colony-forming units, after 2 days incubation at 30 ºC. The growth of yeast transformed with the empty vector (control) was considered as 100%. From the dose–response curves obtained, EC_50_ (half maximal effective concentration) values were determined for the tested compounds.

## Conclusion

In this study, we conducted the phytochemical analysis of the aerial parts of *P. grandidentatus*. From this plant, two extraction methods of **Roy** were explored, namely, CO_2_ supercritical extraction (3.6% w/w of yield) and ultrasound-assisted acetonic extraction (2.3% w/w of yield). **Roy** concentration in each extract was quantified by HPLC–DAD. The study of the CO_2_ supercritical extract revealed the presence of 42.7 μg mg^−1^ of **Roy** (yield of 0.13%), while the acetonic extract contained 55.2 μg mg^−1^ of **Roy** (yield of 0.15%), indicating higher efficiency of the latter. Additionally, the acetonic extraction method was optimized performing three cycles of 30 min each, improving the isolation yield.

For esterification using benzoyl chloride and acetic anhydride as reagents, various conditions were investigated. Benzoylation of **Roy** yielded mono-benzoylated **Roy-12-Bz** and di-benzoylated **RoyBz**, with the 12-OH position being the most reactive. Similarly, **Roy-12-Ac** and **RoyAc** were successfully prepared, though it was not possible to obtain selective functionalization on the 6-OH. The desired derivatives were obtained using mild conditions with overall good yields (33–86%). Double derivatization required higher temperature (50 °C), excess of reagents, and a longer reaction time, yielding compounds with 28–79% yield. Additionally, treatment of crude mixtures in presence of acidic (5% HCl) or alkaline (5% NaOH) work-up conditions led to degradation of the products and re-obtainment of the starting material **Roy**.

The cytotoxic potential of **DeRoy**, **Roy**, and its derivatives **DiRoy**, **RoyBz**, **Roy-12-Bz**, **RoyAc**, **Roy-12-Ac**, and **RoyPr** was evaluated against a panel of breast cancer cell lines, with **RoyBz** showing the most promising activity. Results suggest that derivatization at position C-12 did not offer advantages over di-esterification in the positions C-12 and C-6, and over the parent compound **Roy**. Additionally, the presence of aromatic groups favored cytotoxicity, while **DiRoy**, with three hydroxyl groups, exhibited significantly weaker effects suggesting the importance of the acetoxy group in position C-7 for the cytotoxic activity against breast cancer cell lines.

A small library of royleanones were evaluated as PKC-α, βI, δ, ε, and ζ activators. **DeRoy** exhibited efficacy across all PKC isoforms surpassing the positive controls (PMA and ARA). **RoyPr**, showed promising activation of PKC-δ but not PKC-ζ, indicating subtle structural changes can influence isoform selectivity. Molecular docking analysis suggests differences in binding poses between PKC-α and PKC-δ, emphasizing the role of microenvironmental factors in isoform specificity. These findings underscore the complexity of PKC modulation and highlight the need for further exploration of structural determinants in PKC isoform selectivity.

## Data Availability

The datasets generated and analyzed during the current study are not publicly available due to requirements of the ongoing PhD work but are available from the corresponding author upon reasonable request.
